# Evaluation of penehyclidine for prevention of post operative nausea and vomitting in patients undergoing total thyroidectomy under total intravenous anaesthesia with propofol-remifentanil

**DOI:** 10.1186/s12871-022-01857-5

**Published:** 2022-10-14

**Authors:** Ting Lu, Rongrong Li, Jiacheng Sun, Jing Chen

**Affiliations:** Department of Anesthesiology and Perioperative Medicine, First Affiliated Hospital with Nanjing Medical University, Nanjing, 210029 Jiangsu China

**Keywords:** Penehyclidine, TIVA, Postoperative nausea and vomiting, Total thyroidectomy

## Abstract

**Backgroud:**

Postoperative nausea and vomiting (PONV) is one of the most common complications after total thyroidectomy under general anesthesia. Total intravenous anesthesia (TIVA) has been documented to prevent PONV in patients undergoing total thyroidectomy. Penehyclidine, an anticholinergic agent with an elimination half-life of over 10 h, is widely used as premedication to reduce glandular secretion. This study aimed to explore the preventative effects of penehyclidine with propofol-remifentanil-TIVA to single-TIVA on PONV in patients undergoing total thyroidectomy.

**Methods:**

A total of 100 patients scheduled for total thyroidectomy were randomly assigned to either the penehyclidine group (*n* = 50) or TIVA group (*n* = 50). Propofol and remifentanil were was used for TIVA in all patients. No patients who received premedication. Patients were administrated with either 5 ml of normal saline or 0.5 mg of penehyclidine soon after anesthesia induction. The incidence of nausea and vomiting, the severity of nausea, the requirement of rescue antiemetics, and adverse effects were investigated during the first 24 h in two time periods (0–2 h and 2–24 h).

**Results:**

The overall PONV incidence during the 24 h after surgery was significantly lower in the penehyclidine group compared with the TIVA group (12% vs 36%, *P* < 0.005). Besides, the incidence of nausea and the incidence of vomiting were significantly lower in the penehyclidine group compared with the TIVA group at 2–24 h after surgery. However, there was no significant difference between the two groups at 0–2 h after surgery.

**Conclusions:**

Administration of penehyclidine under TIVA with propofol-remifentanil is more effective for prevention of PONV than TIVA alone, especially 2–24 h after total thyroidectomy.

**Trial registration:**

https://www.chictr.org.cn/edit.aspx?pid=132463&htm=4 (Ref: ChiCTR2100050278, the full date of first registration: 25/08/2021).

## Background

The thyroid is located near the esophagus and trachea. Within the proximity are relatively important blood vessels and nerves, including the internal jugular artery and vein, recurrent laryngeal nerve, and superior laryngeal nerve. Therefore, patients are prone to various complications after thyroid surgery, among which postoperative nausea and vomiting (PONV) is the most common complication. The occurrence of PONV in thyroid surgery is associated with many risk factors. The common risk factors include female, nonsmokers, a history of PONV or motion sickness, and the use of opioids [1]. PONV increases the risk of aspiration of gastric contents, suture dehiscence, postoperative bleeding, and airway obstruction by hematoma, which may affect the surgical treatment and postoperative recovery time [2]. The incidence of PONV after thyroid surgery is reported to be 60–80% when no prophylactic antiemetic is administered [3, 4].

TIVA has been documented to prevent PONV after various surgeries [3]. In addition, TIVA has been recommended by recent guidelines as an equivalent intervention for the prevention of PONV, comparable to one single antiemetic [4]. However, the use of TIVA with a single-drug pharmacological prophylaxis such as 5-HT3 antagonists did not decrease PONV sufficiently across previous study [5].

Many drugs have been tried for the prevention of PONV, and anticholinergics has been shown to be effective in this regard [6–8]. The recommended anticholinergic agent to prevent PONV is transdermal scopolamine patch [9, 10]. Other anticholinergic drugs for preventing PONV, such as glycopyrrolate and atropine, have been shown to be ineffective [11].

Currently, the effect of penehyclidine, a new anticholinergic agent with a long elimination half-life, has been proved to mitigate PONV in patients after strabismus surgery [12]. However, no data is used on penehyclidine as an antiemetic against PONV in patients undergoing thyroid surgery receiving TIVA. This study was to compare the preventative effects of penehyclidine under TIVA with propofol-remifentanil to single-TIVA on PONV in patients undergoing total thyroidectomy.

## Methods

The study was approved by the Review Board of the First Affiliated Hospital with Nanjing Medical University (number 2019-SR-238) and the trial was registered at https://www.chictr.org.cn/edit.aspx?pid=132463&htm=4(Ref:ChiCTR2100050278,the full date of first registration: 25/08/2021). Written informed consent was obtained from all the subjects or their legal guardians. A total of 181 subjects, who were American Society of Anesthesiologist (ASA) physical status I-II and aged 24 ~ 64, scheduled for total thyroidectomy with central compartment node dissection years were screened. Exclusion criteria were body mass index of more than 30 kg/m^2^, smoking history, history of PONV or motion sickness, severe cardiopulmonary disease, history of hepatic or renal disease, medication with steroids, or cognitive impairment. The subjects requiring radical neck dissection were excluded because their operation time would be longer than those of simple total thyroidectomy. All subjects were in a euthyroid state at the time of surgery. The same surgeon performed the thyroid surgery using similar techniques.

The patients were randomly allocated to the TIVA group or penehyclidine group by computer-generated randomization in a 1:1 ratio. All patients did not receive premedication before surgery. Each patient was monitored with electrocardiography, non-invasive blood pressure monitor, and pulse oximetry. General anesthesia was induced with propofol (Corden Pharma S.P.A, Caponago, Italy) 1.5–2.5 mg/kg and fentanyl (Humanwell Healthcare CO.,LTD., China) 2 μg/kg, and orotracheal intubation was performed after administration of cisatracurium (Jiangsu Hengrui Medicine CO.,LTD., China) 0.15 mg/kg. Anaesthesia was maintained with propofol infusion at a rate of 60–200 μg·kg^− 1^·min^− 1^, and remifentanil (Humanwell Healthcare CO.,LTD., China) infusion at a rate of 0.1–0.15 μg·kg^− 1^·min^− 1^ without the use of inhalational anaesthetics. Lactic Ringer’s solution was infused at a rate of 10–15 ml/kg/h throughout the surgery. Mechanical ventilation was used with a tidal volume of 6–8 ml/kg and a frequency of 10–12 beats per minute to keep end tidal CO_2_ at 35–45 mmHg throughout the surgery.

Fresh gas was adjusted to 1 L oxygen to 1 L air with an oxygen concentration of about 60%. In the PACU, residual muscle relaxation was not antagonized by neostigmine and atropine.

The anesthesia nurse who prepared the drug/placebo mixtures according to the group assignment was not involved in this study. After anesthesia induction, 0.5 mg penehyclidine (Avanc Pharmaceutical CO.,LTD., China) in 5 ml or an equal volume of 0.9% normal saline (Shanghai Baxter Medical Supplies CO.,LTD., China) was administrated immediately in the penehyclidine and TIVA group, respectively.

A resident blinded to the treatment evaluated nausea and its severity, vomiting, postoperative pain, the requirement of rescue antiemetic, use of additional analgesics, and side effects at 2 and 24 h after surgery.

Patients were instructed before the operation. The intensity of nausea was based on a 10-point numerical rating scale (NRS: 0 = no nausea at all to 10 = the most severe nausea). The severity of nausea was finally described by NRS scores (mild 1–3, moderate 4–6, severe 7–10). The severity of pain was measured on a 10-point visual analog scale (VAS) (0 = no pain; 10 = most severe pain) [13].

The patients who complained of severe nausea and/or vomiting were rescued with 3 mg granisetron (Shandong Shenglu Pharmaceutical CO.,LTD., China), and severe pain VAS score of more than 5 was treated with 40 mg of parecoxib (Pharmacia &Upjohn Company LLC, U.S.A).

The sample size was calculated based on the incidence of PONV (40%) with TIVA in the literature reviews [5, 14]. Assuming a 30% reduction in the incidence of PONV in penehyclidine group could be considered clinically significant. The value of α would be 0.05 with a power (1 – β) of 0.8. A total of 36 patients per group were required.

All values are expressed as mean ± standard deviation or number percentage. Continuous variables were compared using the Student’s t-test or Mann-Whitney U test according to the normality. Categorical variables were compared using the Chi-square test or Fischer’s exact test, as appropriate. Ranked data was compared using the Mann-Whitney U test. A *P*-value < 0.05 was considered statistically significant. SPSS software for Windows version 25.0 (IBM Corp., Armonk, NY, USA) was used.

## Result

**Fig. 1:**
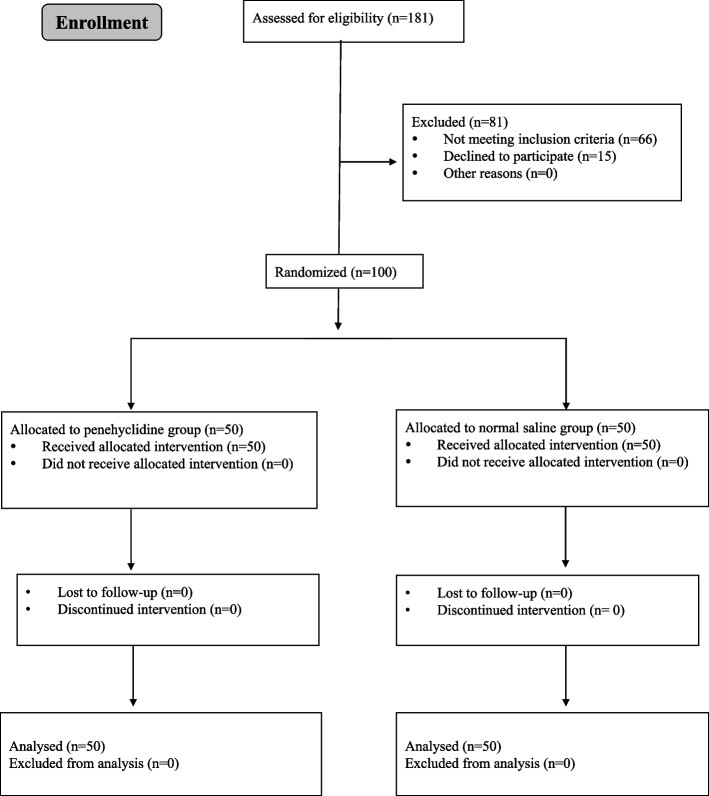
Consort flow diagram of participants

A total of 181 patients were enrolled in this study and 100 patients completed the protocol between December 2019 and January 2021 (Fig. 1). The patient characteristics (including age, gender, body weight), operation data, and fentanyl consumption were statistically similar between two groups (Table 1).

**Table 1:** Patient characteristics and clinical data

	Penehyclidine (*n* = 50)	TIVA (*n* = 50)	*P* value
Age (yr)	42.8 ± 9.6	43.6 ± 10.1	0.590
Gender (M/F)	13/37	17/33	0.762
Body weight (kg)	64.5 ± 11.2	65.3 ± 11.7	0.674
Body height (cm)	165.3 ± 6.9	163.8 ± 7.5	0.473
Duration of surgery (min)	76.6 ± 13.9	75.5 ± 15.5	0.171
Duration of anesthesia (min)	95.9 ± 14.5	95.9 ± 16.7	0.104
Fentanyl consumption (mg)	0.41 ± 0.05	0.42 ± 0.06	0.492
Remifentanil consumption (μg)	580.4 ± 135.9	581.8 ± 129.8	0.431

*TIVA* Propofol-based total intravenous anesthesia. Values are expressed as mean ± SD or ratio. There was no significant difference between two groups

A total of 181 patients were randomly allocated to penehyclidine or TIVA groups. Among them, 66 patients dropped out due to not meeting inclusion criteria and 15 patients declined to participate this study. Therefore, 100 patients were finally analyzed. *N* = 50 patients in TIVA and in penehyclidine group.

**Fig. 2:**
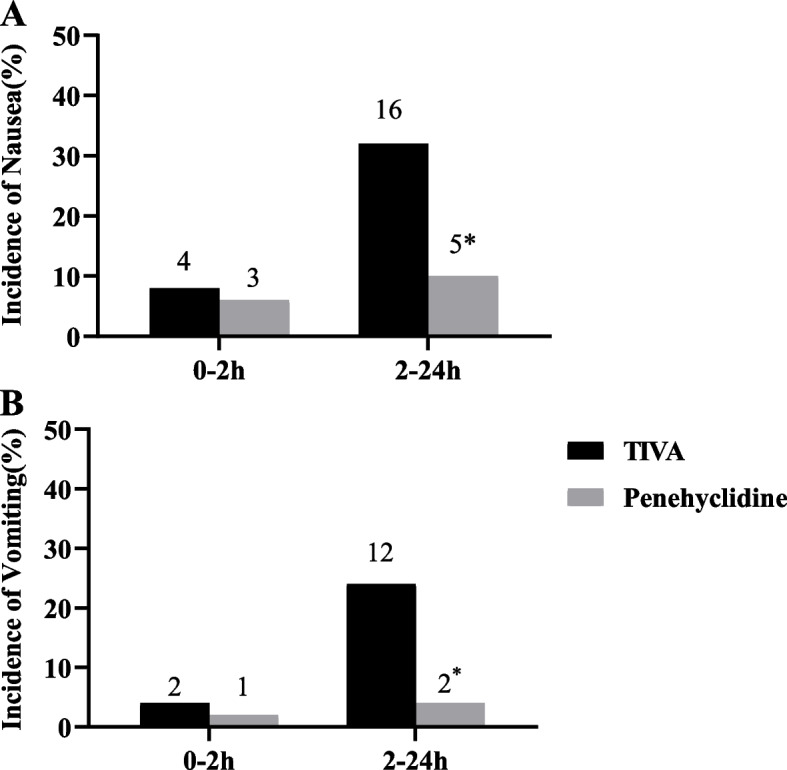
Incidence of nausea (**A**) and vomiting (**B**) in penehyclidine and TIVA groups during 0–2 and 2–24 h after surgery. * *P* < 0.05 compared with the TIVA

**Fig. 3:**
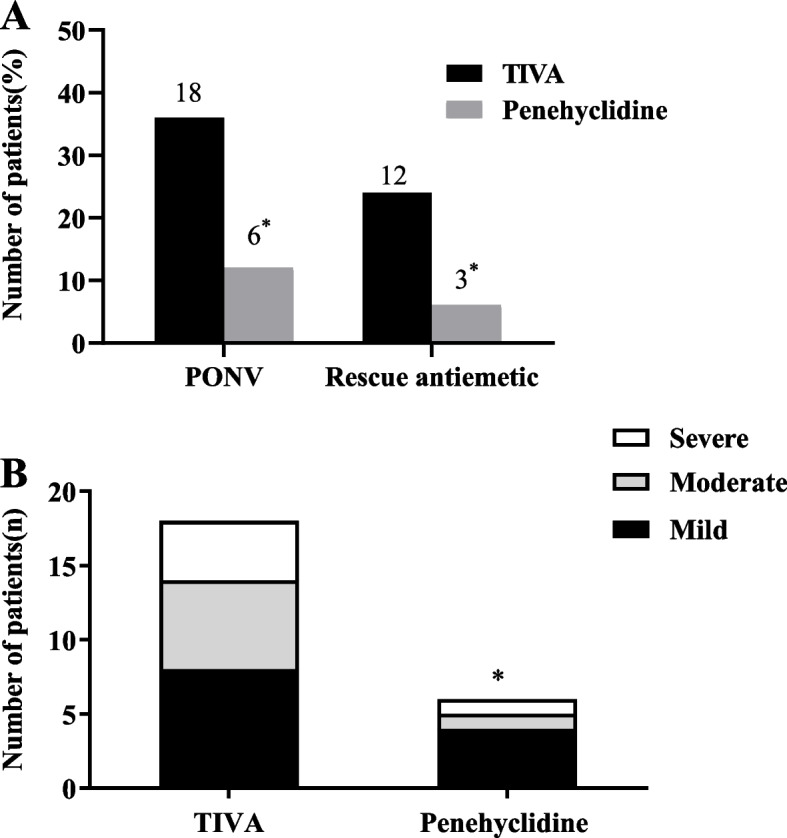
Incidence of PONV and rescue antiemetics (**A**), and severity of nausea (**B**) in penehyclidine and TIVA groups during postoperative 24 h. ^*^ *P* < 0.05 compared with TIVA group

The overall PONV incidence during the 24 h after surgery was significantly lower in the penehyclidine group compared with TIVA group (12% vs 36%, *P* = 0.005; Fig. 3). Besides, the incidence of nausea (10% vs. 32%, *P* = 0.007) and the incidence of vomiting (4% vs. 24%, *P* = 0.009; Fig. 2) were significantly lower in the penehyclidine group compared with the TIVA group at 2–24 h after surgery. However, there was no significant difference between the penehyclidine and TIVA group at 0–2 h after surgery.

The overall PONV incidence 24 h after surgery, proportion of patients who required rescue antiemetic treatments, and severity of nausea were significantly lower in the penehyclidine group than in the TIVA group (6% vs. 24%, *P* = 0.025; *P* = 0.001; Fig. 3).

There were no significant differences in total consumption of fentanyl, VAS pain score and the rescue analgesic requirement during the study period. There were also no significant differences in the incidences of dry mouth, headache and dizziness between the two groups (Table 2).

**Table 2:** Postoperative adverse events

	Penehyclidine (*n* = 50)	TIVA (*n* = 50)	*P* value
Pain score 0–2 h	2.68 ± 0.96	2.66 ± 0.96	0.937
2–24 h	1.48 ± 0.68	1.40 ± 0.70	0.563
Rescue analgesics	4 (8)	4(8)	1.00
Dry mouth	14(28)	8(16)	0.148
Headache	9(18)	8(16)	0.790
Dizziness	10(20)	9(18)	0.799

*TIVA* Propofol-based total intravenous anesthesia. Values are presented as mean ± SD or number (%) of patients

## Discussion

PONV is one of the most common complications and the most unpleasant aspect after thyroid surgery under general anesthesia. This complication can delay patient discharge from the hospital and increase the cost of care [15, 16]. Thyroid surgery is specifically associated with a high incidence of PONV. The main cause of the high incidence of PONV after thyroid surgery is not thoroughly clear, but it is thought to result from the hyperextension of the neck and strong vagal stimulation [17]. Hyperextension of neck posture may lead to cerebral blood flow disorders which can cause central nausea and vomiting [18]. And strong vagal stimulation by surgical handling of neck structures may exacerbate the incidence of PONV [19, 20].

Muscarinic receptors are involved in PONV by various mechanisms [21, 22]. Golding et al. [23] Reported that M3 and M5 acetylcholine receptors have been shown to reduce motion sickness, a risk factor of PONV. The vestibular system is densely packed with M1 receptors, and cholinergic transmission from the vestibular nuclei to the central nervous system centers and from the medullary reticular formation to the vomiting center is blocked by anticholinergics. Additionally, in thyroid surgery, surgical handling of neck structures strongly stimulates the vagus nerve in neck [24]. Anticholinergics have been shown to be effective to prevent PONV, and the recommended anticholinergic drug is scopolamine [9, 11]. Due to its short half-life, scopolamine is used as a transdermal patch before surgery.

Penehyclidine (2-hydroxyl-2-cyclopentyl-2-phenyl-ethoxy) is a new long-acting anticholinergic drug with anti-muscarinic and anti-nicotinic activities that has potent central and peripheral anticholinergic activities. It is widely used as a pharmacologic agent for organic phosphorus poisoning and preoperative medication, but its effect on PONV is unclear. Penehyclidine has a greater selectivity for muscarinic 1(M1) and muscarinic 3 (M3) subtypes of acetylcholine receptors but no effect on muscarinic 2 (M2) subtype of acetylcholine receptors [25]. Given its mechanism of action, its effect on PONV was to be expected. Previous reports showed that penehyclidine mitigated the incidence of PONV in patients after strabismus surgery [12] and gynecological laparoscopic surgery [26]. In our study, we also found that penehyclidine reduced PONV in patients undergoing thyroid surgery. In these surgeries, the draw reaction is a routine operation which may be related to the higher incidence of PONV.

The previous studies have demonstrated that propofol prevent the incidence of PONV during the early 0–2 h postoperative period rather than late [5, 27], which is consistent with the results of our study. Our analysis shows that patients receiving TIVA had a higher incidence of PONV in the late postoperative phase, starting at 2 h after surgery.

TIVA has been documented to prevent PONV after thyroid surgery. Apfel et al. [28]suggested that the risk factors for early PONV (< 2 h) and late PONV (2–24 h) are different, and inhalation or TIVA is not a risk factor for late PONV. A longer-acting antiemetic drug may be necessary to prevent late PONV after TIVA [27, 29]. Penehyclidine has a longer elimination half-life (10.4 ± 1.22 h) than that of ondansetron (3.5 h) or granisetron (4.9 h) or ramosetron (9 h) [30, 31]. Our study suggests that penehyclidine effectively reduced the late incidence of PONV (2–24 h) than early PONV (0–2 h) in patients after TIVA. The use of TIVA with a single-drug pharmacological prophylaxis did not decrease PONV acrossing to the previous study [5].However, However, in our study, the use of TIVA with penehyclidine decreases PONV sufficiently and mitigates the severity of nausea after thyroid surgery. Administration of penehyclidine after anesthesia induction can be widely used as a pharmacologic agent on PONV in patients undergoing thyroid surgery.

The main side effects of penehyclidine are dry mouth, headache and central anticholinergic syndrome. In the present investigation, none of the patients presented with central anticholinergic syndrome, and there was no difference between the two groups in the incidence of dry mouth and headache. These may possibly be explained by the use of a limited dose of 0.5 mg penehyclidine.

Potential risk factors contributing to PONV, such as etomidate and neostigmine were not administrated in the thyroid surgery [32]. The gender of the patients was mostly female, which was consistent with previous reports (female-to-male ratio 2–4:1) [33]. Besides, we strictly performed the randomization and double-blinded technique during the study.

A limitation of the current study should be noted. We anticipated a reduction of about 30% between the two groups before our study. However, the actual reduction in overall PONV incidence was 24% (36% in TIVA group vs 12% in penehyclidine group, *P* = 0.005) during the 24 h after surgery. But the relative reduction rate of 30–40% in general PONV study is considered clinically relevant, the acquisition of a relative risk reduction of 67% in our study can be considered clinically significant [24, 34]. However, this operation was performed as a TIVA with propofol-remifentanil infusion. How high if using inhalational agents is unknown. Further studies are needed to research penehyclidine in more patients at more diverse surgical settings using different anesthetic techniques.

## Conclusions

In conclusion, administration of penehyclidine after total intravenous anaesthesia with propofol-remifentanil significantly reduces the incidence of PONV especially 2–24 h after thyroidectomy. Penehyclidine, a widely used preoperative anticholinergic agent, can be considered a as an effective anti-emetic protector in patients undergoing thyroid surgery.

## Data Availability

The datasets are not publicly available due to the stipulations of ethics committee to protect individual privacy of patients but are available from the corresponding author on reasonable request.

## Abbreviations

PONV: Postoperative nausea and vomiting
TIVA: Propofol-based total intravenous anesthesia
5-HT3: 5-hydroxytryptamine
